# Essential medicines containing ethanol elevate blood acetaldehyde concentrations in neonates

**DOI:** 10.1007/s00431-016-2714-x

**Published:** 2016-03-21

**Authors:** H. C. Pandya, H. Mulla, M. Hubbard, R. L. Cordell, P. S. Monks, S. Yakkundi, J. C. McElnay, A. J. Nunn, M. A. Turner, on behalf of the ESNEE consortium

**Affiliations:** Department of Infection, Immunity and Inflammation, University of Leicester, University Road, Leicester, LE1 9HN UK; Department of Pharmacy, University of Hospitals Leicester NHS Trust, Leicester, UK; Neonatal Unit University of Hospitals Leicester NHS Trust, Leicester, UK; Department of Chemistry, University of Leicester, Leicester, UK; Department of Pharmacy, Queen’s University Belfast, Belfast, UK; Alder Hey Children’s NHS Foundation Trust, Liverpool, UK; Department of Women’s and Children’s Health, Institute of Translational Medicine, University of Liverpool, Liverpool, UK

**Keywords:** Infants, Drug excipients, Alcohol

## Abstract

Neonates administered ethanol-containing medicines are potentially at risk of dose-dependent injury through exposure to ethanol and its metabolite, acetaldehyde. Here, we determine blood ethanol and acetaldehyde concentrations in 49 preterm infants (median birth weight = 1190 g) dosed with iron or furosemide, medicines that contain different amounts of ethanol, and in 11 control group infants (median birth weight = 1920 g) who were not on any medications. Median ethanol concentrations in neonates administered iron or furosemide were 0.33 (range = 0–4.92) mg/L, 0.39 (range = 0–72.77) mg/L and in control group infants were 0.15 (range = 0.03–5.4) mg/L. Median acetaldehyde concentrations in neonates administered iron or furosemide were 0.16 (range = 0–8.89) mg/L, 0.21 (range = 0–2.43) mg/L and in control group infants were 0.01 (range = 0–0.14) mg/L. There was no discernible relationship between blood ethanol or acetaldehyde concentrations and time after medication dose.

*Conclusion*: Although infants dosed with iron or furosemide had low blood ethanol concentrations, blood acetaldehyde concentrations were consistent with moderate alcohol exposure. The data suggest the need to account for the effects of acetaldehyde in the benefit-risk analysis of administering ethanol-containing medicines to neonates.

**Table:** 

**What is Known:**
• *Neonates are commonly treated with ethanol-containing medicines, such as iron and furosemide.* • *However, there is no data on whether this leads to appreciable increases in blood concentrations of ethanol or its metabolite, acetaldehyde.*
**What is New:** • *In this study, we find low blood ethanol concentrations in neonates administered iron and/or furosemide but markedly elevated blood acetaldehyde concentrations in some infants receiving these medicines.* • *Our data suggest that ethanol in drugs may cause elevation of blood acetaldehyde, a potentially toxic metabolite.*

## Introduction

Preterm neonates are often chronically treated with oral liquid formulation medicines that contain ethanol as an excipient [12, 13, 20, 22]. In this context, ethanol is used as an organic phase co-solvent. It also has antimicrobial preservative properties [12, 13, 20, 22]. Whittaker et al. have previously ethanol exposure in preterm neonates treated with oral liquid drugs, in particular furosemide and iron [2], and raised concerns that a chronic drug therapy could cause an acute and/or chronic ethanol toxicity [12, 22].

There is little data on the effects of ethanol exposure in preterm infants. In infants and children, ethanol poisoning may cause hypoglycaemia, cardio-respiratory depression and seizures [16]. Exposure to ethanol in utero is associated with fetal alcohol syndrome [2]. The pathophysiology of fetal alcohol syndrome is complex. It involves exposure of tissues to ethanol and acetaldehyde, a highly reactive and toxic by-product of ethanol metabolism during vulnerable periods of organ development [2]. Since pre- and post- natal organ development correlates with age, it follows that neonates born prematurely and exposed to ethanol could be at risk of ‘ex utero fetal alcohol syndrome’. Moreover, as ethanol effects in adults correlate with blood ethanol concentrations [24], it is likely that adverse effects of ethanol in preterm infants will also correlate with blood ethanol concentrations.

In the UK, oral liquid iron and furosemide medicines contain 0.3 (300 mg/100 ml) and 7.5 % (7500 mg/100 ml) ethanol, respectively. In our neonatal unit, if clinically indicated, preterm infants receive between 1 and 2.5 ml of iron solution preparation daily (ethanol 3–7.5 mg) and up to 1 ml/kg furosemide daily (ethanol 75 mg/kg). To test the hypothesis that administration of these drug medicines to preterm neonates elevates blood ethanol concentrations, we conducted a population study of blood ethanol and acetaldehyde concentrations in infants treated with oral iron and furosemide.

### Methods

Seventy-six babies receiving iron and/or furosemide preparations and 11 control subjects were enrolled into this study. The study received ethical approval from NRES Committee North West – Greater Manchester North Rec, ref no: 11/NW/0665. The investigation was conducted in line with the Declaration of Helsinki. All children were recruited from the neonatal units within University Hospitals of Leicester NHS Trust. Infants on oral iron and/or furosemide therapy were eligible as were infants who were on no ethanol-containing medicines (control group). Neonates were excluded if parents or guardians refused or were unable to give valid consent. Neonates were prescribed iron or furosemide based on clinical indications and according to unit protocols. Control babies were not on any medication containing ethanol. All oral medicines were administered with feeds (milk) and given via a naso/orogastric tube or mixed into a bottle with milk. Demographic and clinical details including birth weight, gestation, drugs doses and drug dose times were recorded on a case report form (CRF).

### Sample collection and analysis

Ethanol and acetaldehyde were measured in blood samples using a static headspace gas chromatography coupled mass spectrometry (GC-MS) as previously described by our group [5]. The technique allows quantification of ethanol and acetaldehyde in micro-volumes of blood using n-propanol as an internal standard. The linearity of the method was established over the range 0.1–100 mg/L (*R*^2^ > 0.99): limit of detection = 0.1 mg/L, lower limit of quantification = 0.5 mg/L [5]. Intra- and inter-day precision and accuracy for ethanol and acetaldehyde were 20 and ≤25 %, respectively. Most babies receiving iron and/or furosemide had blood ethanol concentrations measured on more than one occasion. The median number of samples obtained from study babies was 4 (range 1–22). Seven babies in the control group had blood ethanol concentrations measured on one occasion only. The timing of blood samples in relation to administration of iron or furosemide was entirely based on the clinical needs of the child since study blood was opportunistically scavenged from samples taken for routine blood tests.

### Data analysis

Data from each CRF, comprising the iron and furosemide dosing pattern and blood-sampling times were reconciled with blood ethanol and acetaldehyde concentrations. The data were collated and analysed using Excel and GraphPadPrism software. Ethanol and acetaldehyde concentrations were related to either iron or furosemide administration based on the medicine the child received prior to the blood sample. Differences in blood ethanol and acetaldehyde concentrations between groups were compared using an unpaired Student’s *t* test. Linear correlation analysis was performed using Pearson product moment correlation coefficient.

## Results

### Patient demographics

Seventy-six babies receiving iron and/or furosemide and 11 babies receiving neither (control group) were recruited into the study. Forty-nine infants receiving iron and/or furosemide and 11 babies in control provided at least 1 blood sample for analysis. Demographic and clinical data of babies providing 1 or more blood samples are presented in Tables 1 and 2. In comparison to infants receiving iron and/or furosemide, control group babies were significantly heavier (median birth weight 1.92 vs.1.19 kg) and older at birth (median gestational age at birth 231 vs. 193 days) and had significantly greater post-menstrual weight (median weight 2.05 vs. 1.87 kg) at recruitment. No baby in either group had clinical or biochemical evidence of hepatic or renal dysfunction.

**Table 1:** Demographics, liver and renal function tests of neonates recruited into the study and providing blood samples. Results are from analysis of serum

	Iron and/or furosemide neonates median (range) *N* = 49	Control group neonates median (range) *N* = 11
Birth weight	1.19 (0.63–2.38)	1.92 (0.61–3.93)
Gestational age at birth (days)	193 (171–245)	231 (171–289)
APGAR score	8 (4–10)	8 (4–10)
Weight at recruitment (kg)	1.87 (1.06–3.34)	2.05 (0.52–3.93)
Post-menstrual age at recruitment (days)	247 (190–326)	239 (174–293)
Post-natal age at recruitment (days)	29 (10–94)	10 (2–27)
Sampling times after last dose (hours)	30.8 (0–321.6)	Not applicable
Dose of ethanol (mg)/dose of iron or furosemide	7.24 (2.27–78.42)	Nil
Duration in study (days)	28 (0–101)	1.5 (1–5)
Albumin (g/dl)	44 (18–44)	34 (20–62)
Alkaline phosphatase (IU/L)	442 (124–1312)	313 (27–532)
Alanine transaminase (IU/L, ALT)	18 (10–120)	5 (0.4–10)
Bilirubin (umol/L)	32 (2–118)	Not determined
Creatinine (umol/L)	30 (19–135)	49 (20–63)
Urea (mmol/L)	4.2 (0.4–11.8)	3.9 (1.2–6.9)

**Table 2:** Demographics of neonates grouped according to ethanol-containing medicine

	Iron only neonates (median and range)	Iron and furosemide (median and range)	Furosemide only (median and range)
Gestational age at birth (days)	200 (175–245)	192 (171–227)	200 (185–209)
Post-natal age at recruitment (days)	30 (10–82)	27 (12–52)	28.5 (22–94)
Weight at recruitment (kg)	2.04 (1.06–3.34)	1.68 (1.04–3.28)	1.58 (1.15–3.09)
Ethanol dose/dose of medicine (mg/kg)	1.1 (0.7–3.9)	6.7 (1.4–10.9)	11.8 (9.1–14.5)

### Blood ethanol and acetaldehyde concentrations in neonates receiving iron and/or furosemide

**Fig. 1:**
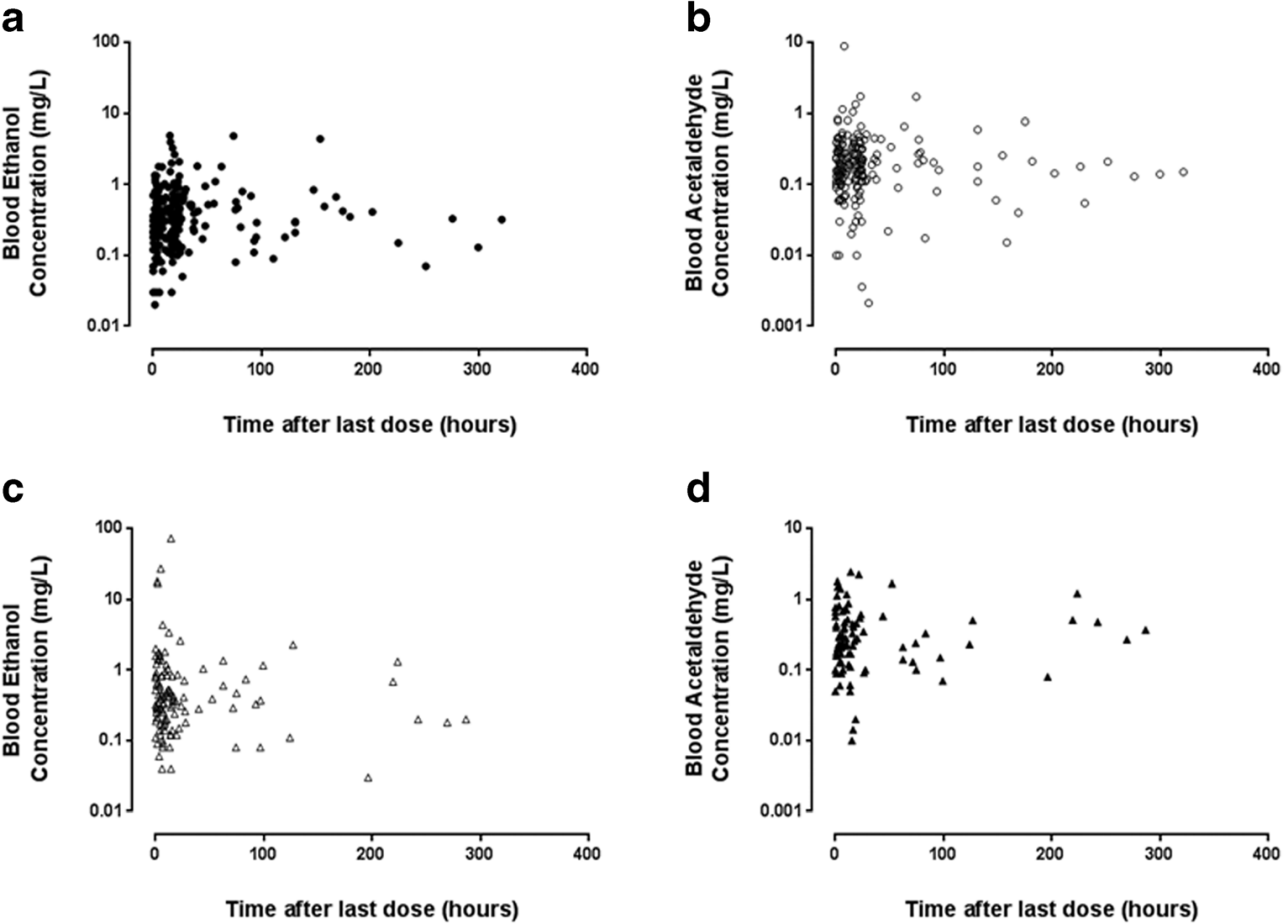
Blood concentrations of ethanol (**a**,**c**) and acetaldehyde (**b**,**d**) plotted against time. Blood concentrations are related to dosing with iron (**a**,**b**) or furosemide (**c**,**d**) according to the medication the child received prior to blood sampling. There is no discernible correlation between blood ethanol (*white circle* and *black circle*) or blood acetaldehyde (*white triangle* and *black triangle*) concentrations and time after administration of iron or furosemide

Blood ethanol and acetaldehyde concentrations were measured in 348 blood samples from babies receiving either iron only (*N* = 35 babies), iron and furosemide (*N* = 10 babies) or furosemide only (*N* = 4 babies). Ethanol concentrations above 1 mg/L were found in 67 blood samples (20 % of total) from 26 infants; 2 babies (2 blood samples) had blood ethanol concentrations above 20 mg/L. Median ethanol concentrations in the iron (*N* = 231 blood samples) and the furosemide (*N* = 117 blood samples) groups were 0.33 (range = 0–4.92) and 0.39 (range = 0–72.8) mg/L, respectively. There was no discernible relationship between blood ethanol concentrations and time after dose of an ethanol-containing drug medicine (Fig. 1). However, sub-analysis showed that infants with ethanol >5 mg/L received an ethanol-containing medicine within 24 h of obtaining the blood sample. In control group infants (*N* = 14 time points), ethanol concentrations ranged between 0.03–5.4 mg/L (median = 0.15 mg/L). Three blood samples from 3 infants (28 % of total) in this group had ethanol concentrations above 1 mg/L. There was no significant difference in blood ethanol concentrations in infants receiving ethanol-containing medicines and control group infants (*P* > 0.05).

Two hundred seventeen blood samples from 42 infants administered iron and/or furosemide blood acetaldehyde concentrations were above 0.1 mg/L. Fifteen babies (*N* = 117 blood samples) had acetaldehyde concentrations above 0.5 mg/L. Median blood acetaldehyde concentrations in the iron (*N* = 231 samples) and the furosemide (*N* = 117 samples) groups were 0.16 (range = 0–8.89) and 0.21 (range = 0–2.43) mg/L, respectively. There was no discernible relationship between blood acetaldehyde concentration and time after dose of iron or furosemide (Fig. 1). In control group infants (*N* = 14 blood samples), the median blood acetaldehyde concentrations was 0.01 (range = 0–0.14) mg/L. Two infants from this group had acetaldehyde concentrations above 0.1 mg/L. Infants who received furosemide prior to blood sampling had significantly higher blood acetaldehyde concentrations than control group infants (*P* = 0.02). There was no significant difference in acetaldehyde concentrations comparing infants administered iron prior to blood sampling and control group infants (*P* > 0.05).

### Correlation between blood ethanol and acetaldehyde concentrations

**Fig. 2:**
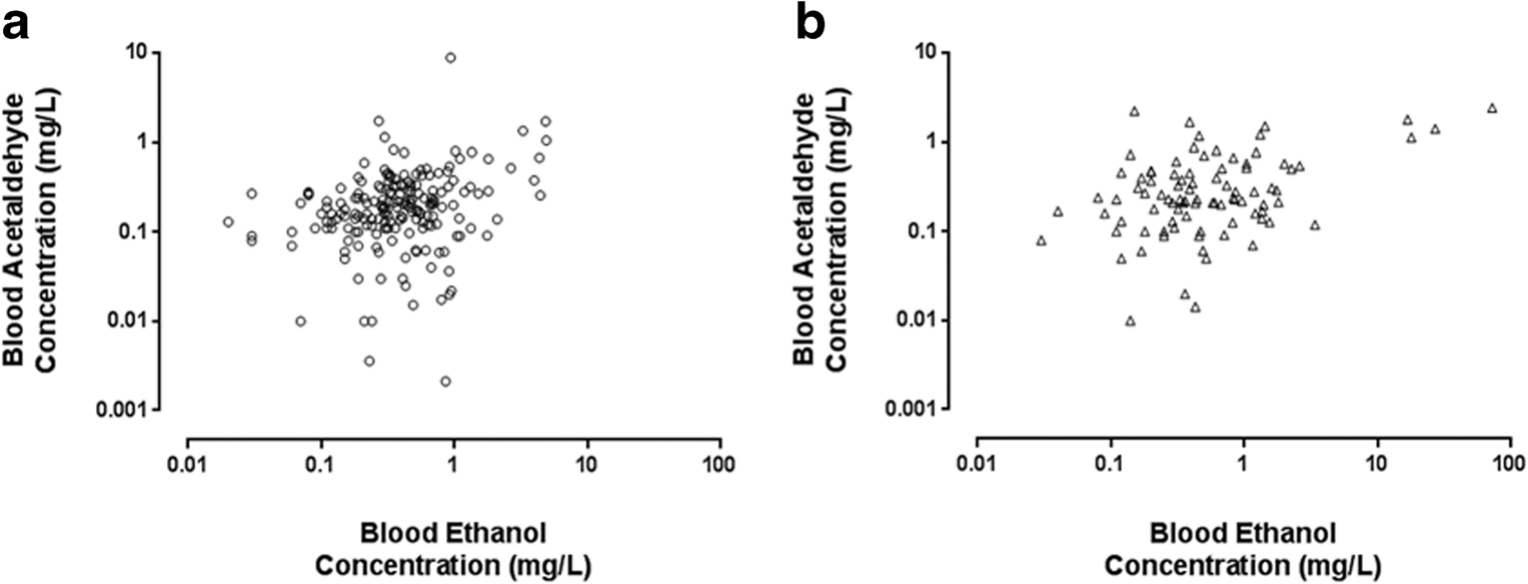
Plots of blood ethanol vs. blood acetaldehyde concentrations in neonates receiving iron and/or furosemide. Blood concentrations are related to dosing with iron (**a**) or furosemide (**b**) according to the medication the child received prior to blood sampling. For both iron and furosemide groups, there was a weak but significant correlation between blood ethanol and blood acetaldehyde concentrations. Pearson product moment coefficient, *R* ^2^ = 0.05 and *R* ^2^ = 0.34 for iron and furosemide groups, respectively, *P* < 0.01 for both

There was a weak linear correlation between blood ethanol and blood acetaldehyde concentrations in infants who received iron and in infants who received furosemide before blood sampling (see Fig. 2), Pearson product moment coefficient, *R*^2^ = 0.05 and *R*^2^ = 0.34 for iron and furosemide, respectively, *P* < 0.01 for both. There was a no significant linear correlation between blood ethanol and blood acetaldehyde in control group infants.

## Discussion

This study shows that preterm babies administered iron and/or furosemide preparations formulated with ethanol have a broad range of blood ethanol and acetaldehyde concentrations. Relative to blood ethanol concentrations used to define drink-driving offences in many countries (>500 mg/L mainland Europe; >800 mg/L UK and USA), blood ethanol concentrations in preterm infants were low. However, blood acetaldehyde concentrations in this population were higher than baseline concentrations (<0.1 mg/L) recorded in otherwise healthy, alcohol abstinent, adults.

There is little data on baseline blood ethanol concentration in ethanol-abstinent preterm neonates or young children. In healthy alcohol-abstaining adults, Sprung et al. found mean blood ethanol concentration of 0.27 mg/L with a range between 0.2–0.8 mg/L [18]. Although blood concentrations greater than 0.8 mg/L have been recorded in teenagers, mean ethanol concentrations (approximately 0.3 mg/L) in this age group are not different to those recorded in adults [9, 14, 18]. The median and range of blood ethanol concentrations in infants receiving iron/furosemide are higher and broader than those determined in teenagers or adults in earlier studies. Over 50 % of ethanol-exposed infants had at least 1 blood concentration greater than 1 mg/L, and 1 neonate had a blood concentration of nearly 80 mg/L. Despite these differences, our overall conclusion is that treatment of preterm infants with medicines containing between 0.3–7.5 % (300–7500 mg/100 ml) ethanol does not result in toxicologically relevant systemic concentrations of ethanol in blood. Although fewer control group infants were found to have blood ethanol greater than 1 mg/L than those receiving iron and/or furosemide (28 vs. 53 %, respectively), median blood concentrations in these two groups were not significantly different.

The ethanol concentrations found in this study lend support to the concept that neonates have minimal systemic exposure to ethanol following enteral administration of ethanol-containing medicines at the presently reported dosing levels. This observation allays concerns raised in our previous study in which ethanol exposure was extrapolated from medicines, including iron and furosemide, routinely administered on a neonatal unit [22]. The most likely explanation is efficient and rapid first-pass metabolism of ethanol to acetaldehyde.

Blood acetaldehyde concentrations in infants administered iron and/or furosemide were higher than those in control group infants. The implication of elevated blood acetaldehyde concentrations in infants receiving ethanol-containing medicines are unclear as little is known about the sources, kinetics and effects of acetaldehyde in this age group or even older children. In healthy adults, blood acetaldehyde concentrations correlate with dose of ethanol and aldehyde dehydrogenase (ALDH) genotype [1, 6]. Whilst we did not genotype any of the infants recruited in this study, blood acetaldehyde concentrations did correlate with blood ethanol concentrations in neonates receiving iron and/or furosemide (Fig. 2) but not in control group infants (data not shown). These observations support an ‘ethanol dose’ effect on blood acetaldehyde concentrations in preterm infants. However, despite differences in administered doses of ethanol (see Table 2), there was no difference in acetaldehyde concentrations between the iron and furosemide groups. Since no infant was co-administered an acetaldehyde dehydrogenase inhibitor (e.g., metronidazole), one explanation is that measured acetaldehyde levels reflect cumulative doses of ethanol, which may not have been significantly different between the iron and furosemide groups. Ontogenic differences in acetaldehyde-metabolising capacity could also account for the lack of dose-effect. Nonetheless, our data suggest that the acetaldehyde to acetate conversion pathway becomes overwhelmed at relatively low doses of ethanol.

Presently, it is only possible to speculate about the clinical effects of elevated blood acetaldehyde concentrations in preterm babies. In adults, signs and symptoms of elevated blood acetaldehyde concentrations resemble those of ethanol intoxication. In ‘aldehyde syndrome’ of adults, elevated blood acetaldehyde due to ethanol ingestion is associated with facial flushing, tachycardia, reduced psychomotor function, muscle weakness and sleeplessness [9] These symptoms occur at blood acetaldehyde concentrations ranging between 0.4 and 2.6 mg/L [1, 6, 9]. Thus, some of the infants in the present study had acetaldehyde concentrations consistent with symptoms associated with the aldehyde syndrome.

The relatively large numbers of patients recruited into this study can be mostly attributed to parents finding opportunistic collection and use of scavenged blood acceptable, as others have reported [3, 4, 8]. Whilst acceptable to parents, opportunistic sampling confers limitations on data analysis and interpretation. For example, if sampling time points do not cover the whole dosing period, it may not be possible to describe or model drug/excipient absorption and metabolism accurately.

As blood samples were processed and managed in clinical chemistry laboratories prior to being frozen for study assays, there is a possibility that some ethanol and acetaldehyde, both volatile at room temperature, could have evaporated from blood during processing of samples. Hence, if anything, the data presented may underestimate blood ethanol and acetaldehyde concentrations in babies recruited into the study. However, in-house in vitro studies indicate that recovery of ethanol and acetaldehyde from blood samples allowed to equilibrate with room air for 60 min was >95 % [5]. The small numbers of babies in the control group and demographic difference between control group infants and those receiving iron/furosemide are noteworthy, and we cannot exclude the possibility that these babies inadvertently ingested ethanol as some infants were fed with breast milk.

High blood acetaldehyde concentrations associated with administration of oral liquid iron and/or furosemide suggest that preterm neonates receiving these medicines are at risk of excipient-related neurotoxicity [6, 24]. However, there is insufficient data to quantify the impact of treatment with these medicines in this patient population. In the UK, oral liquid furosemide is generally administered to a small group of infants with heart failure or chronic lung disease of prematurity [17, 19]. Neurocognitive outcomes in these two patient groups are recognised to be poor [11, 21]. In contrast, oral liquid iron is widely used to prevent and treat anaemia in neonates [10]. Hence, many more infants are likely to be exposed to ethanol through iron therapy than furosemide administration. Both iron and furosemide are listed as essential medicines by the World Health Organisation [10]. How our observations relating to use of oral liquid iron and furosemide in the UK extrapolate to other countries, other drugs and to older infants is unclear as drug prescribing and drug formulation practices vary considerably across the world [23]. The risks of medicines formulated with potentially toxic excipients such as ethanol are recognised hurdles to improving health outcomes for children globally [7, 15, 23]. In this context, our findings reinforce the global need to develop drugs and drug delivery systems suited to the needs of children [23].

Conclusion: This is the first study to determine blood concentrations of ethanol and its metabolite acetaldehyde in neonates receiving ethanol-containing medicines as part of routine care. Neonates were found to have low blood ethanol concentrations following treatment with iron and furosemide. However, in some neonates, blood acetaldehyde concentrations were markedly elevated several hours post-dose. We infer that ethanol in drugs may cause elevation of blood acetaldehyde, a metabolite that has the potential to harm neonates. Hence, acetaldehyde may be a bigger concern than ethanol in infants administered these medicines. In principle, babies with genotypes associated with reduced ethanol- or acetaldehyde-metabolising capacity should be treated with ethanol-free drug preparations. Whilst our observations imply a ‘minimal ethanol in medicines’ policy may be the only way of preventing neonates being inadvertently exposed to acetaldehyde through administration of medicines, presently, the risk of harms from acetaldehyde will need to be balanced against the likelihood of benefits from the active ingredient.

## Abbreviations

ALDH: Aldehyde dehydrogenase
CRF: Case report form
ESNEE: European Study of Neonatal Exposure to Excipients
GC-MS: Gas chromatography coupled mass spectrometry
